# Ancient visual channels have a causal role in arithmetic calculations

**DOI:** 10.1038/s41598-021-02260-9

**Published:** 2021-11-23

**Authors:** William Saban, Asael Y. Sklar, Ran R. Hassin, Shai Gabay

**Affiliations:** 1 Department of Psychology and the Institute of Information Processing and Decision Making (IIPDM), University of Haifa, Haifa, Israel; 2 Department of Psychology, Hebrew University, Jerusalem, Israel; 3 Department of Psychology, and the Center for the Study of Rationality, Hebrew University, Jerusalem, Israel; 4 Department of Psychology and Helen Wills Neuroscience Institute, University of California, Berkeley, CA 94720 USA

**Keywords:** Psychology, Human behaviour, Cultural evolution

## Abstract

Humans exhibit complex arithmetic skills, often attributed to our exceptionally large neocortex. However, the past decade has provided ample evidence that the functional domain of the subcortex extends well beyond basic functions. Using a sensitive behavioral method, for the first time, we explored the contributions of lower-order visual monocular channels to symbolic arithmetic operations, addition and subtraction. The pattern of results from 4 different experiments provides converging evidence for a *causal relation* between mental arithmetic and primitive subcortical regions. The results have major implications for our understanding of the neuroevolutionary development of general numerical abilities–subcortical regions, which are shared across different species, are essential to complex numerical operations. In a bigger conceptual framework, these findings and others call for a shift from the modal view of the exclusive role of the neocortex in high-level cognition to a view that emphasizes the interplay between subcortical and cortical brain networks.

## Introduction

Humans exhibit exclusive complex arithmetic abilities, which are often attributed to the exceptional enlargement of humans’ neocortical regions during evolution. Scientific investigations that examine arithmetic reasoning tasks have long emphasized the importance of neocortical regions. Yet, subcortical regions have been overlooked and even neglected by the neuroscientific literature in general, and by most studies that examine arithmetic behaviors in particular. Hence, the possible involvement of primitive subcortical regions, which are shared across different species, remains an open question. To examine this question, we explored whether lower visual channels (subcortical and V1 regions) have a functional role in humans’ arithmetic reasoning (calculation and comparison processes).

Most studies have demonstrated the involvement of fronto-parietal^1–7^ neocortical regions in arithmetic abilities. Meta-analyses of functional magnetic resonance imaging (fMRI) studies demonstrated activity of neocortical regions such as the inferior parietal lobule and prefrontal cortices during arithmetic calculations^7^. Developmental neuroimaging studies indicated that over the course of childhood, children develop numerical skills (e.g., increasingly precise representations of numerical values). Interestingly, the intraparietal sulcus has a developmental trajectory that is a key neural correlate of numerical cognition^8^. Recent studies, using different methods such as primate neurophysiology, developmental neuropsychology, and human neuroimaging, indicate that numerical abilities in general (both symbolic and nonsymbolic) have a neocortical substrate involving prefrontal and parietal regions^4^. One of the most influential models of number-related processes—the Triple Code model^3^—suggests that numerical information is represented mainly by the neocortex.

To conclude, due to the fact that symbolic arithmetic is a cultural product and a high-level cognitive function, and in accordance with the complexity of arithmetic calculations, the literature has long emphasized the involvement of mostly neocortical regions in symbolic arithmetic. It appears that areas of the neocortex play a critical role in humans’ arithmetic abilities. However, as in the case of many other high-level cognitive functions^9–14^, humans’ arithmetic abilities might be based on evolutionarily ancient brain circuits.

While the literature provides sophisticated models for the involvement of cortical networks in cognition, scientific investigations have long overlooked subcortical regions, and their functional role in cognition is still unknown. The neuroscientific literature underrepresents subcortical mechanisms, likely due to a “corticocentric bias” that overemphasizes the role of cortical regions in cognition. This overlooking of subcortical regions was previously noted and demonstrated in a review by Parvizi^15^, who coined the term “corticocentric” to describe the conceptual bias that masks the possibility that “higher” functions might also depend on “lower” structures, and thus generates negative implications for subcortical regions in current cognitive neuroscience research. In his paper, Parvizi suggested that some neuroscientific methods suffer from a bias of overemphasizing cortical involvement in cognitive processes. Several methods such as electroencephalography, magnetoencephalography, near-infrared spectroscopy, optical imaging, and transcranial magnetic stimulation cannot be used to unravel the possible role of subcortical structures. In addition, in clinical neuroscience, studies examining patients with neurological and psychiatric disorders are mainly focused on the involvement of cortical regions^15^. In addition to Parvizi’s claims regarding a methodological bias, one of the most commonly used methods for exploring the neural substrates of cognition also suffers from a “corticocentric” tendency. Although fMRI studies provided us insights regarding the potential involvement of subcortical regions, one methodological limitation of fMRI is its reduced ability to detect activations in subcortical structures^16^. Not only are many of the structures quite small, but scanning protocols are rarely optimized for detecting subcortical activation. Hence, most fMRI studies are prone to overemphasizing the cortex involvement in cognitive processes. To summarize, the different methods used for studying neural substrates of cognition in general and arithmetic in particular are focused mainly on the neocortex and are limited in their ability to infer the possible involvement of primitive subcortical mechanisms.

In addition to the “corticocentric” bias, from an evolutionary perspective, brain organization is subject to strong anatomical and connectional constraints, inherited from evolution, under which new abilities find their “neuronal niche,” i.e., a set of circuits that are sufficiently close to the required function^17^. When an evolutionarily novel function (e.g., symbolic arithmetic) invades an older one (e.g., basic numerical abilities), its prior neural constraints might exert a powerful influence on brain organization.

Humans rapidly enumerate collections of objects, add them, and compare their numerosity. It was suggested that these basic numerical abilities underlie our comprehension of symbolic numerals such as Arabic numerals^18^.For example, the Approximate Number System (ANS), an evolutionarily and ontogenetically ancient system^19^, allows approximate representation of numbers. Research has found that the ANS, which is an elementary form of numerical intuition, can predict subsequent mathematical scores at school, and suggested that ANS is a building block of later mathematical ability^20^ (see also^21^).

Most pertinent to the present study, using a stereoscope, a recent study^22^ demonstrated the involvement of monocular subcortical regions in discriminating numerosity in larger ratios (4:1 or 3:1), but not in smaller ones (2:1) nor in Arabic numeral comparison tasks. This recent finding is limited to basic numerical discrimination abilities, such as the rudimentary and nonsymbolic skills exhibited by infants, which do not involve symbolic computations. Although humans’ arithmetic abilities may be phylogenetically novel, it is possible that primitive subcortical regions may serve as a phylogenetic bridge to these higher arithmetic abilities.

Furthermore, we assume that numerical abilities can be traced through the evolution of species^23^. It is reasonable to presume that during evolution, numerical abilities were necessary for survival in an ever-changing environment. Indeed, a wide range of organisms, which possess varied neural substrates (e.g., fish, anurans, honeybees, parrots, spiders), have nonsymbolic and symbolic numerical abilities such as discriminating larger quantities, distinguishing between ratios, identifying and serially ordering Arabic numerals, and more^24–29^. For instance, a range of numerical behaviors were demonstrated in birds, including discrimination^30^ and even arithmetic in new born chicks^31^. Interestingly, the domestic chick, tested in monocular conditions, can successfully perform in numerical tasks such as mapping number to space^32^.

The ability to represent, discriminate, and perform arithmetic operations on discrete quantities has been documented in many species of different taxonomic groups, both vertebrates and invertebrates^19,23^. Overall, studies suggest the existence of an ancient ANS which has been conserved through evolution and allows species to approximately estimate the numerosity of stimuli^19,33^. A recent review provided a comparative portrait of the neurobiology of numerical cognition and suggested that numerosity might be processed “without the need to speculate on complex networks or sophisticate brains.”^33^.

To summarize, many organisms, which are evolutionarily distinct from humans, do possess numerical abilities that enable them to survive through evolution. The primitive subcortical regions that humans share with different species are involved in basic numerical abilities, and such fundamental skills are essential for the emergence of more advanced arithmetic abilities. Hence, the outstanding question is whether these primitive subcortical brain regions are involved not only in rudimentary nonsymbolic numerical skills, but also in complex symbolic arithmetic calculations. The present study will explore if the origins of human complex arithmetic abilities might be founded upon evolutionarily ancient brain circuits.

In order to explore the contribution of subcortical regions to arithmetic abilities, we used a psychophysical method that allows one to differentiate between higher (mostly neocortical) and lower (mostly subcortical) visual channels’ involvement. Visual input is monocularly segregated until it reaches striate and extrastriate regions^34,35^. Thus, subcortical visual channels are monocularly segregated while higher cortical visual channels are mostly insensitive to the eye-of-origin of the visual information. As such, dividing the visual input between the eyes is a manipulation that influences mostly subcortical brain regions (V1 and lower visual channels). If these regions are functionally involved in a specific cognitive task, then dividing the visual information between them will affect the performance in this task. By using a stereoscope, one can present different visual information to each eye separately, thereby dissociating the contribution of monocular (mostly subcortical) versus binocular (mostly cortical) visual channels in different cognitive processes^10,36,37^. If monocular channels have a functional role in a cognitive process, then segregating the visual information to different eyes should affect performance.

**Figure 1:**
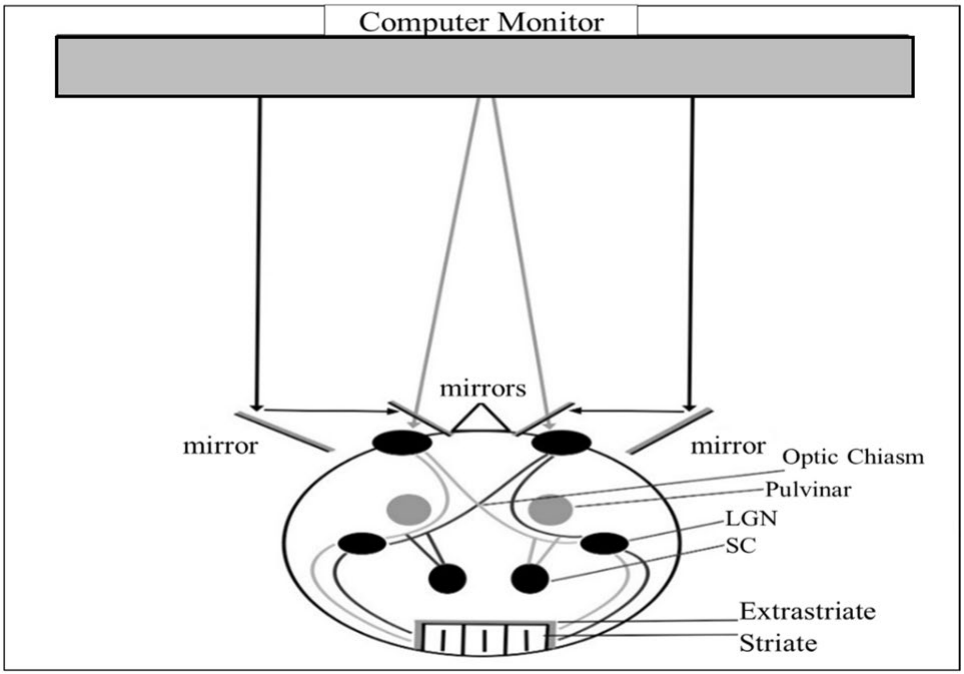
Schematic illustration of the experimental apparatus and visual pathways from the eyes to the brain. Each side of the computer monitor provided visual information to a different eye. From the eye, the visual information passes first through monocularly segregated subcortical regions (grey lines: left eye; black lines: right eye). This information is then projected to the lateral geniculate nucleus (LGN) and subsequently reaches striate and binocular extrastriate regions.

**Figure 2:**
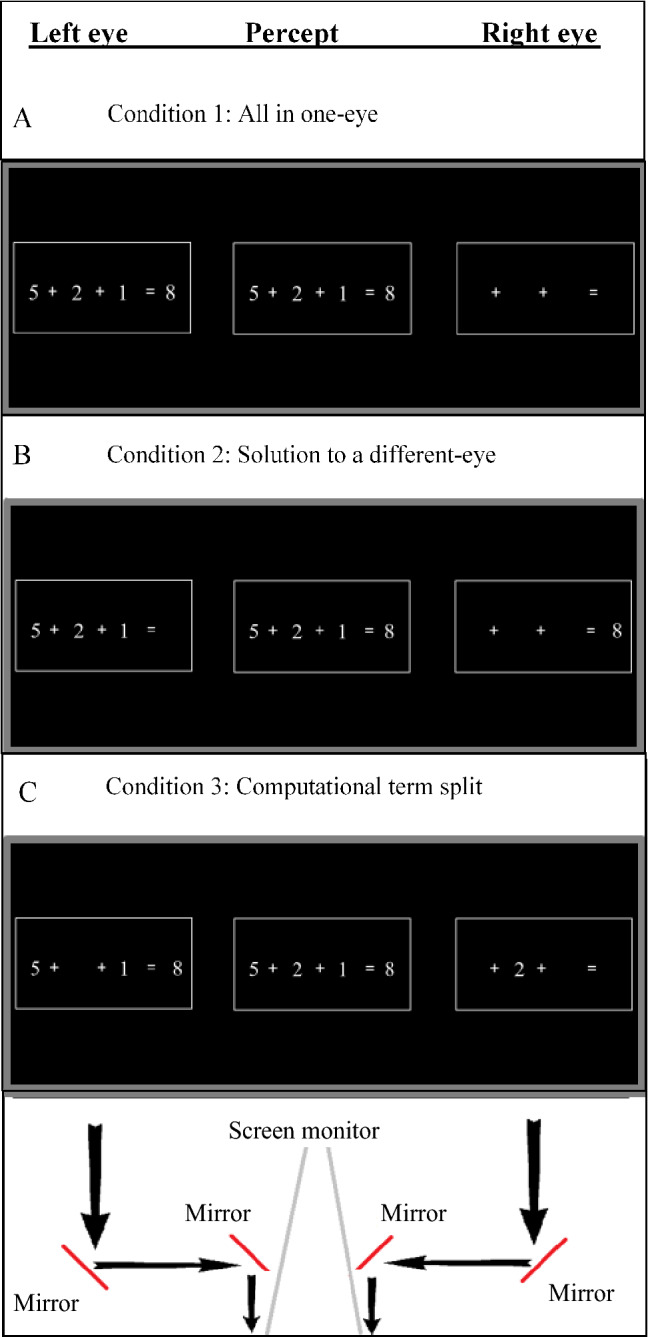
The verification task in which participants were asked to solve equations (by subtraction and addition) composed of an arithmetic problem of three numbers and a solution (e.g., 5 + 2 + 1 = 8). The three numbers and the equation’s solution were either presented: (**A**) All in one-eye or (**B**) Solution to a different-eye: arithmetic problem to one eye and the solution to the other eye or (**C**) Computational term split: one of the numbers to one eye and the other two numbers and the solution to the other eye.

The present study challenges the scientific view that argues for the exclusive involvement of neocortical regions in arithmetic calculations. Specifically, we argue that humans’ lower visual channels (subcortical and V1 regions) not only passively channel information, but also have a functional role in symbolic arithmetic calculations. In all the experiments, we use a stereoscope (Fig. 1) to examine whether monocular channels can modulate the performance of multi-digit subtraction and addition calculations. The task employed in our experiments, that of evaluating whether a presented equation is correct or incorrect (e.g., 9 – 4 – 2 = 3), involves two separate processes. One is to calculate the arithmetic problem on the left-hand side of the equal sign. The other is to compare the solution of the arithmetic problem to the number presented on the right-hand side of the equal sign. In order to examine the contribution of monocular channels to the calculation vs. comparison processes, we manipulated the eye to which the different numbers in an equation were presented (the “eye-of-origin manipulation”). In one condition, the arithmetic problem (i.e., the numbers on the left-hand side of the equal sign) and the solution (i.e., the number on the right-hand side) were both presented to the same eye (the “All in one-eye” condition; see Panel A of Fig. 2). In a second condition, the arithmetic problem and the solution were each presented to a different eye (the “Solution to a different-eye” condition; see Panel B of Fig. 2). In a third condition, one of the numbers in the arithmetic problem was presented to a different eye than the other numbers in the equation (“Computational term split” condition; see Panel C of Fig. 2). If monocular channels are involved in the calculation process, then performance should be hampered in the “Computational term split” condition compared to the other conditions. If monocular channels are involved in the comparison process, then performance should be hampered in the “Solution to a different-eye” condition compared to the “all in one-eye” condition.

In Experiment 1, participants performed a verification task in which they were asked to evaluate equations composed of an arithmetic problem of three numbers (united by subtraction or addition operators) and a solution (e.g., 9 – 5 – 3 = 1) as either correct or incorrect. The equations were displayed in one of the three eye-of-origin conditions described above: (1) All in one-eye or (2) Solution to a different-eye or (3) Computational term split. Before each experiment, the stereoscope apparatus was calibrated for each participant individually to ensure the perceptual fusion of the images presented in all the conditions. In Experiment 2, in order to examine the influence of the typical visual presentation (which might involve memory-based and perceptual processes), the equations were presented in vertical alignment (from top to bottom) instead of the canonical horizontal alignment. In Experiments 1 and 2, we also manipulated the incorrect answers’ distance from the correct answer since responding to an answer at a small distance from the correct answer requires exact calculation, whereas responding to an answer at a bigger distance relies on rough estimation to some extent. As different operators (e.g., subtraction and addition) may involve different brain regions^7,38^ we examined the involvement of monocular channels both in addition and subtraction abilities.

In Experiment 3, we replaced the solution with a second arithmetic problem, which requires a calculation of its own (e.g., 6 + 3 = 4 + 5), in order to examine whether each monocular channel can be involved in a different calculation process independently. Finally, in Experiment 4 we explored the limits of the involvement of monocular channels in arithmetic calculations. As subcortical regions are relatively primitive brain regions, we hypothesized that the involvement of the even more complex and evolutionarily later decimal system in arithmetic calculations would limit the usefulness of subcortical regions. Hence, we examined the influence of the eye-of-origin manipulation in equations that involve the decimal system, which include double-digit numbers that might require even more complex arithmetic abilities (e.g., 60 – 2 – 7 = 51).

## Experiment 1

### Results

In Experiment 1, we carried out a three-way analysis of variance (ANOVA) with condition (All in one-eye, Solution to a different-eye, Computational term split), operator (addition, subtraction), and distance (zero, small, big) as within-subject factors, and reaction time (RT) as the dependent variable.

Since in some comparisons we predicted the null hypothesis (no difference between the “Solution to a different-eye” and the “All in one-eye” conditions), we also calculated Bayes factors (BF_01_). The BF_01_ provides information about the ratio between the strength of evidence of the null hypothesis (quantified in the BF numerator) and the strength of evidence of the alternative hypothesis (quantified in the BF denominator)^39^. As previously suggested^40^, the BF_01_ values can be classified by the following: 1–3 anecdotal, 3–10 medium, 10–30 strong, and 30–100 very strong evidence for the null hypothesis (H0). By contrast, the BF_10_ values indicate evidence for H1. The BFs were calculated using JASP (https://jasp-stats) with the default Cauchy prior (0.707).

**Figure 3:**
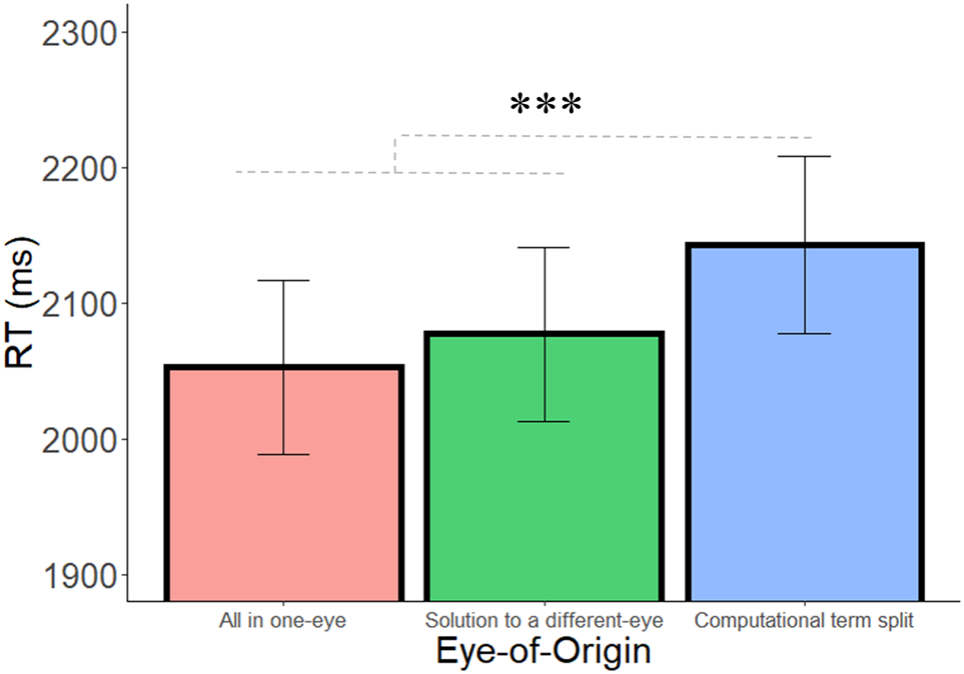
RT as a function of eye-of-origin in Experiment 1. Error bars = SEM. *** = *p* < .001.

The main effects of eye-of-origin, operator, and distance were significant (F(2,88) = 9.03, *p* < 0.001, ${\upeta }_{\mathrm{p}}^{2}$= 0.17; F(1,44) = 48.26, *p* < 0.001, ${\upeta }_{\mathrm{p}}^{2}$= 0.52; F(2,88) = 77.77, *p* < 0.001, ${\upeta }_{\mathrm{p}}^{2}$= 0.64, respectively). Most importantly, in order to examine the contribution of monocular visual channels to the arithmetic calculation process, we compared the Computational term split condition, in which one of the numbers was presented to a different monocular channel, to the two conditions in which the entire arithmetic problem was presented to a single monocular channel. Follow-up planned comparisons analyses of the eye-of-origin effect revealed that the RT was significantly slower in the Computational term split condition, compared with the average of the two other conditions (F(1,44) = 13.17, *p* < 0.001, ${\upeta }_{\mathrm{p}}^{2}$= 0.23, BF_10_ = 77.51; Fig. 3; Accuracy: 93%, 92.7%, 92.9% in the computational term split, solution to a different-eye and the all in one-eye conditions, respectively), which were not significantly different from one another (F(1,44) = 1.74, *p* = 0.19, ${\upeta }_{\mathrm{p}}^{2}$= 0.04, BF_01_ = 2.75). RT was significantly faster when the solution was presented to a different eye compared to the Computational term split condition (F(1,44) = 10.47, *p* = 0.002, ${\upeta }_{\mathrm{p}}^{2}$= 0.19, BF_10_ = 28). In addition, RT was significantly faster in the All in one-eye condition compared to the Computational term split condition (F(1,44) = 11.90, *p* < 0.001, BF_10_ = 48.00). The pattern of results demonstrates that presenting one number of the arithmetic problem (on the left-hand side of the equation) to a different eye than the rest of the numbers delays performance, which suggests that monocular channels have a functional role in the calculation process.

Additionally, the two-way interactions between eye-of-origin and operator and the two-way interaction between eye-of-origin and distance were not significant (F(2,88) = 1.12, *p* > 0.25; F(4,176) = 0.14, *p* > 0.25, respectively). However, the interaction between operator and distance was significant (F(2,88) = 4.98, *p* = 0.008, ${\upeta }_{\mathrm{p}}^{2}$=0.10). Further analyses revealed that there is a bigger difference between subtraction and addition at a big distance compared with the averaged zero and small distances (F(1,44) = 7.68, *p* = 0.008). Zero and small distances were not significantly different from one another (F(1,44) = 0.95, *p* > 0.25). The three-way interaction was not significant (F(4,176) = 0.68, *p* > 0.25).

To conclude, these findings suggest that monocular channels have a functional role in the calculation process, yet there is no evidence for its involvement in the comparison process.

## Experiment 2

### Results

In Experiment 1, the pattern of results demonstrated that lower monocular channels have a functional role in symbolic multi-digit arithmetic calculations. The lack of difference in performance between the solution to a different-eye condition and the all in one-eye condition indicates that monocular channels are not involved in the comparison process.

When acquiring arithmetic skills, children mostly encounter mathematical equations presented horizontally (from left to right). In order to examine the influence of this typical visual presentation (which might involve memory-based and perceptual processes), and to dissociate it from more pure and abstract arithmetic calculation abilities, in Experiment 2 the mathematical equations were presented in a less canonical, vertical alignment (from top to bottom). Other than that, Experiment 2 was identical to Experiment 1.

**Figure 4:**
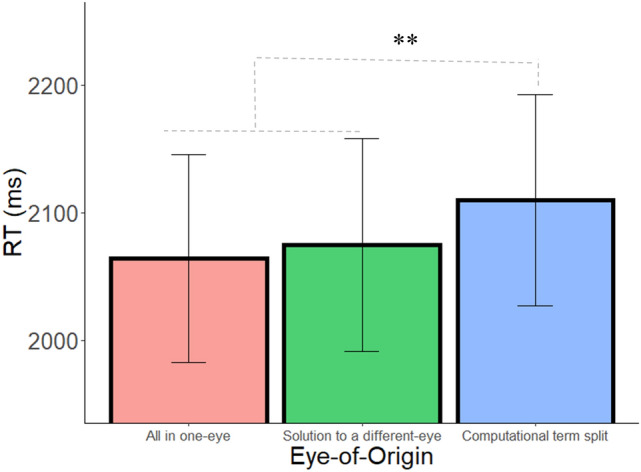
RT as a function of eye-of-origin when equations were vertically aligned. Error bars = SEM. ** = *p* < .005.

We carried out the same analyses as in Experiment 1. The pattern of results in this experiment replicated the pattern observed in Experiment 1. As before, the main effects of eye-of-origin, operator, and distance were significant (F(2,64) = 3.17, *p* = 0.04, ${\upeta }_{\mathrm{p}}^{2}$= 0.09; F(1,32) = 25.18, *p* < 0.001, ${\upeta }_{\mathrm{p}}^{2}$= 0.44; F(2,64) = 54.78, *p* < 0.001, ${\upeta }_{\mathrm{p}}^{2}$= 0.63, respectively). Most importantly, follow-up planned comparisons analyses of the eye-of-origin effect revealed that the RT was significantly slower when one of the numbers in the arithmetic problem was presented to a different eye compared with the average of the two other conditions (F(1,32) = 9.38, *p* = 0.004, ${\upeta }_{\mathrm{p}}^{2}$=0.22, BF_10_ = 17.53; see Fig. 4; Accuracy: 96.3%, 95.7%, 95.8% in the computational term split, solution to a different eye, and all digits to the same eye conditions, respectively), which were not significantly different from one another (F(1,32) = 0.24, *p* > 0.25, BF_01_ = 4.8). When the solution was presented to different eye, RT was significantly faster than when one of the numbers of the arithmetic problem was presented to different eye (F(1,32) = 6.16, *p* = 0.01, ${\upeta }_{\mathrm{p}}^{2}$= 0.16, BF_10_ = 5.15). Similarly, RT was significantly faster in the All in one-eye condition compared to the Computational term split condition (F(1,32) = 5.34, *p* = 0.02, ${\upeta }_{\mathrm{p}}^{2}$= 0.14, BF_10_ = 3.66).

In addition, the two-way interaction between eye-of-origin and operator and the interaction between eye-of-origin and distance were not significant (F(2,64) = 0.10, *p* > 0.25; F(4,128) = 1.46, *p* = 0.21, respectively). The operator and distance interaction was significant (F(2,64) = 13.64, *p* < 0.001, ${\upeta }_{\mathrm{p}}^{2}$= 0.29). Further analyses indicated a bigger difference between subtraction and addition at a big distance compared with both the zero and small distances (F(1,32) = 25.52, *p* < 0.001; F(1,32) = 7.00, *p* = 0.012, respectively), which were also significantly different from one another (F(1,32) = 7.16, *p* = 0.01). The three-way interaction was not significant (F(4,128) = 0.15, *p* > 0.25).

To conclude, replicating the pattern of results of Experiment 1, the current experiment also demonstrated that the Computational term split condition delays performance. This was evident even though equations were presented in vertical alignment, reducing the possible influence of other cognitive processes that might facilitate the verification of equations (e.g., memory-based and perceptual processes that might be involved in basic pattern recognition). This allows one to dissociate pattern recognition processes from pure and symbolic arithmetic calculation abilities.

## Experiment 3

### Results

In Experiment 3, we wanted to examine whether each monocular channel can be involved in a different calculation process independently. We examined this by presenting equations in which a calculation was required on both sides of the equal sign. If each monocular channel can act on its own, and be involved in a different calculation independently, performance should not deteriorate when each eye is presented with a different calculation. If there is an interaction between the two monocular channels, performance should be impaired in this condition. In both cases, we expected to replicate our previous findings, and to observe delayed performance in conditions in which one of the numbers (on either the left- or right-hand side of the equation) is presented to a different monocular channel.

**Figure 5:**
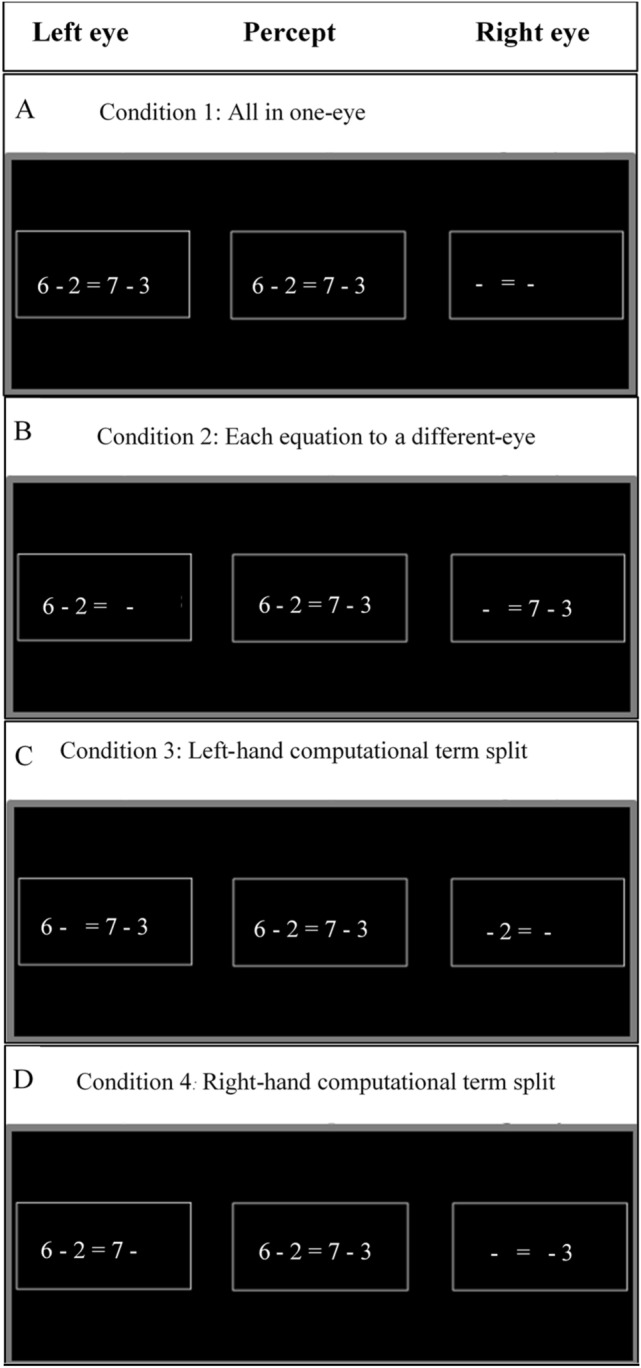
The verification task in which participants were asked to solve equations (by subtraction and addition). The four eye-of-origin conditions were: (**A**) all in one-eye; (**B**) each equation in a different-eye; (**C**) left-hand computational term split; (**D**) right-hand computational term split.

In order to examine whether each monocular channel can be involved in a different calculation process independently, in Experiment 3 the solution component of the equation (which appears on the right-hand side of the equation) was replaced with an arithmetic problem requiring a calculation (e.g., 6 + 3 = 4 + 5). Arithmetic performance in four eye-of-origin conditions was then compared: (1) all in one-eye; (2) each problem in a different-eye; (3) left-hand computational term split (LHCTS), in which a number from the left-hand side of the equation was presented to a different eye; (iv) right-hand computational term split (RHCTS), in which a number from the right-hand side of the equation was presented to a different eye (see Fig. 5). Since distance did not interact with eye-of-origin in the first two experiments, distance was not examined in Experiment 3.

We carried out a two-way ANOVA with condition (All in one-eye, Each problem in a different-eye, LHCTS, RHCTS) and operator (addition, subtraction) as within-subject factors, and RT as the dependent variable.

**Figure 6:**
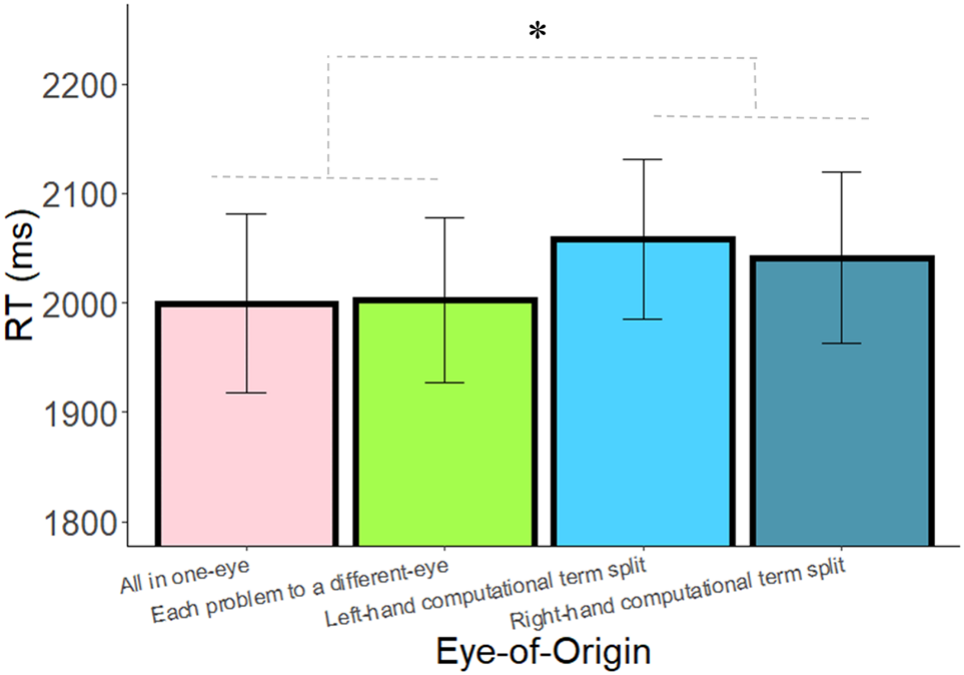
RT as a function of eye-of-origin in Experiment 3. Error bars = SEM. * = *p* < .01.

Most importantly, as in the previous experiment, the main effect of eye-of-origin was significant (F(3,96) = 3.35, *p* = 0.02, ${\upeta }_{\mathrm{p}}^{2}$= 0.09). The main effect of operator and the interaction between eye-of-origin and operator were not significant (F(1,32) = 0.90, *p* > 0.25; F(3,96) = 1.56, *p* = 0.20, respectively). Follow-up planned comparisons analyses of the eye-of-origin effect revealed that the average RTs in the conditions in which one of the numbers was presented to a different eye (LHCTS and RHCTS) were significantly slower compared with the average of the two other conditions (all in one-eye and each problem in a different-eye; F(1,32) = 8.29, p = 0.007, ${\upeta }_{\mathrm{p}}^{2}$= 0.20, BF_10_ = 11.71; see Fig. 6). Further analyses revealed that the comparison between LHCTS and RHCTS was not significant (F(1,32) = 0.65, *p* > 0.25, BF_01_ = 3.97). Similarly, the comparison between the all in one-eye condition and the each equation in a different-eye condition was also not significant (F(1,32) = 0.02, *p* > 0.25, BF_01_ = 5.32).

To conclude, the pattern of results replicated the effects found in Experiments 1 and 2. In addition, when one of the numbers in an arithmetic problem was presented to a different eye, performance was hampered compared with the other two conditions. There was no difference between the condition in which the complete equation was presented to a single eye (which included both arithmetic problems) and the condition in which each eye was presented with a different arithmetic problem. These results suggest that each monocular channel can be involved in a different calculation process simultaneously, such that their functional role in calculation is independent.

## Experiment 4

### Results

In Experiment 4, as subcortical regions are primitive brain regions, it is possible that when more computational power is required neocortical networks get more involved in solving the problems. Hence, in Experiment 4, we examined whether monocular channels are also involved in performing more complex arithmetic calculations that involve two-digit numbers. In Experiment 4 the calculated sums were higher (sums of 20–99) than in all other experiments making the computational demands much higher^41,42^. This is in line with the problem-size effect, that is the increase in response times with the size of the operands^43,44^. This was done by exploring whether the eye-of-origin manipulation influences performance in solving equations whose first (left-most) number is a multiple of 10 ranging between 10 and 90 (e.g., 60 + 3 + 5 = 68). Here we predict a lack of eye-of-origin effect since a greater reliance on neocortical involvement is expected.

**Figure 7:**
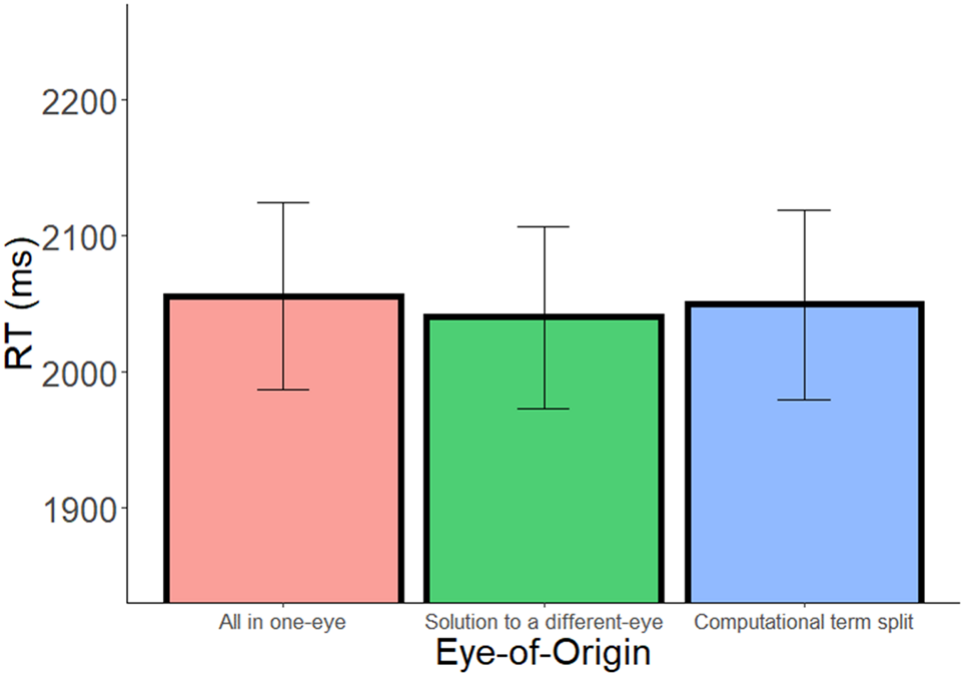
RT as a function of eye-of-origin in Experiment 4. Error bars = SEM. No significant effect of eye-of-origin was found.

As in Experiments 1 and 2, we carried out a two-way ANOVA with condition (All in one-eye, Solution to a different-eye, Computational term split) and operator (addition, subtraction) as within-subject factors, and RT as the dependent variable. Unlike in the previous experiments, the main effect of eye-of-origin was not significant (F(2,62) = 0.47, *p* > 0.25; ${\upeta }_{\mathrm{p}}^{2}$= 0.05, BF_10_ = 0.14; see Fig. 7). The main effect of operator was significant (F(1,31) = 32.79, *p* < 0.001, ${\upeta }_{\mathrm{p}}^{2}$= 0.51), indicating that RT was faster in addition than in subtraction. The eye-of-origin and operator interaction was not significant (F(2,62) = 1.19, *p* > 0.25).

To conclude, the pattern of results demonstrated that the eye-of-origin manipulation does not modulate performance in solving double-digit complex arithmetic problems. This pattern of results does not provide evidence for the functional involvement of monocular channels in solving double-digit equations, in contrast to simpler single-digit arithmetic calculations.

## Discussion

To date, the literature has emphasized the role of humans’ neocortical regions in arithmetic abilities. However, very little is known about the neuroevolutionary development of numerical abilities. A central question addressed by the present study is whether evolutionarily primitive mechanisms and neural substrates are involved in humans’ arithmetic abilities.

The pattern of results arising from our experiments provides evidence for the notion that primitive subcortical regions have a functional role in the performance of symbolic arithmetic calculations. Humans’ primitive subcortical regions are involved in basic numerical abilities^22^, and such rudimentary skills are the building blocks of more advanced arithmetic abilities^18^. In contrast to most of the literature, our findings lend support to the claim that primitive subcortical brain regions that are shared by different species are involved not only in rudimentary numerical skills, but also in arithmetic calculations. The results suggest that neocortical regions are not the only parts of the human brain that are involved in arithmetic, and that a cortico-subcortical loop may supports arithmetic calculations.

Indeed, although most previous literature emphasized the involvement of cortical regions, some studies do support the hypothesis that subcortical regions have a role in general arithmetic processes^45–47^. These studies have demonstrated the relation between subcortical regions (e.g., basal ganglia, thalamus) and general arithmetic processes. Interestingly, one study has shown that stimulation of the thalamus impaired arithmetic processes, and in particular, calculation involving lower numbers. This previous result is in line with our results of Experiment 4 which suggest that the role of subcortical regions is limited to solving lower numbers equations. However, note that all of the above studies have demonstrated the relation between subcortical regions and general arithmetic processes, but none of them, have demonstrated a direct causal role of subcortical regions specifically in arithmetic calculation. In addition, previous studies did not dissociate between different processes involved in solving arithmetic problems such as perceptual, memory retrieval, and comparison processes. In three different experiments, using sensitive-behavioral manipulation, the current pattern of results demonstrated the role of lower visual channels specifically in calculation, and not in other general arithmetic processes.

The current study findings also converge with others which imply that cultural constructions are grounded upon evolutionarily ancient representations, such as space and number ^18,48^. In accordance with previous literature ^48,49^, we suggest that the current fully developed numerical system of humans may have been mediated by the use of more basic nonsymbolic processes (e.g., spatial abilities and conceptual size). Using neurosurgical patients, a recent study has provided evidence of the involvement of non-neocortical regions by demonstrating that single neurons in the human medial temporal lobe encode symbolic and nonsymbolic numerical information^50^. In particular, the study found that numerosity and abstract numerals are encoded by distinct neuronal populations in the medial temporal lobe and suggested that representation of symbolic numerals may evolve from more basic numerosity representations.

Some theoretical accounts even suggest the compositionality of number concepts (e.g., seven is composed of other, smaller numbers). That is, the representation of numbers is itself an arithmetic (set-based) calculation^51^. Accordingly, the involvement of non-neocortical regions in the representation of numbers suggests the involvement of those regions in a more fundamental form of arithmetic calculations. In addition, conceptual size representation is necessary for an organism’s survival in an ever-changing environment (e.g., the ability to determine a predator’s approximate size). Conceptual sizes can thus be viewed as evolutionarily early measurement units of continuous numerical values. That is, conceptual sizes carry long-term knowledge of an object’s size, regardless of the object’s actual retinal size. Thus, they might be considered equivalent to numerals, which (symbolically) denote long-term knowledge of specific quantities. In addition, recent data support the idea that the basic ANS and higher symbolic numerical abilities are intrinsically linked^52^. Congenitally blind and blindfolded sighted participants completed an auditory numerical approximation task and a symbolic arithmetic task. It was found that the precision of approximate number representations was identical across congenitally blind and sighted groups. This finding suggests that the development of the ANS does not depend on visual experience, and that the basic ANS and the higher symbolic numerical abilities are strongly associated^52^.

This proposal is also in line with the recent finding of numerosity representation in human subcortical regions^22^, which indicates the involvement of evolutionarily primitive brain regions in humans’ basic numerical abilities. It is noteworthy that the cited study did not find evidence for subcortical involvement in an Arabic numeral comparison task. This result is in line with our findings of no subcortical involvement in Arabic numeral comparison processes (as we found null effects for the solution to a different-eye condition vs. all in one-eye condition), yet our results do demonstrate a specific subcortical involvement in computational arithmetic processes. Finally, although the modal view in the literature suggests that consciousness is encoded by neocortical regions and is necessary for arithmetic, findings have demonstrated that humans can solve arithmetic equations nonconsciously^53^.

To conclude, the present study pattern of results is in line with recent studies that, in contrast to most of the previous literature, have demonstrated the involvement of noncortical regions in symbolic spatial abilities in humans and fish^54,55^, nonsymbolic and symbolic numerical abilities in humans^22,50^, and symbolic numerical abilities in organisms such as bees^29^. Notably, research on non-verbal organisms has shown that the zebrafish responds to change in visual numerosity^56^, the archerfish have magnitude-related decisions^57^, and newborn chicks can exhibit arithmetic-like behaviors using the ANS^31^.

Recently, using the stereoscopic manipulation, it was demonstrated that the subcortex has a causal role in cognitive transfer of complex cognitive skills in humans^13^. Such cognitive transfer was found for both novel figural-spatial problems (near transfer) and novel subtraction problems (far transfer). These results challenge the exclusive role of the cortex in cognitive transfer as was previously assumed. Most pertinent for the current work, this recent finding demonstrates the direct relations between spatial and arithmetic abilities, but also converge with the notion that the subcortex functionally supports arithmetic^13^.

The present study extends previous literature by demonstrating that subcortical mechanisms support the ability of humans to solve symbolic arithmetic equations. Although nonsymbolic numerical abilities (such as approximation and conceptual size representation) and symbolic numerical abilities might be phylogenetically and ontogenetically distinct, they might have been linked throughout human development. From an evolutionary perspective, one possibility is that basic nonsymbolic numerical abilities may have served as basic units of present-day more formal and complex arithmetic abilities. It is possible that subcortical regions might contribute to numerical representations (such as space and conceptual size) and are used as evolutionary scaffolding for higher arithmetic abilities. We suggest that in a larger conceptual framework, all these novel findings call for a significant update of the modal view of the exclusive role of neocortical mechanisms in higher cognitive functions.

The results have major implications for our understanding of the neuroevolutionary development of numerical abilities. The current findings suggest a parsimonious explanation for higher numerical abilities of different animals, despite their lack of neocortical structures similar to those that have been suggested to support higher cognition in humans. Based on these and other results^9,14,22,55,58–60^ and on evolutionary and developmental theories of the human brain^61^, we propose a general conceptual framework, according to which Subcortical Neural Systems (SNS) may have a functional role in the development and evolution of cognition. According to this framework, since SNS developed early in evolution, and have survived and remained functional up to the present, these systems are essential for cognitive operations that enable organisms to adapt to an ever-changing environment. SNS perform fundamental computations, with the neocortex using these computations to allow the emergence of more complex cognitive abilities. Neocortical regions have access to SNS computations, resulting in a dynamic network that allows more complex cognitive representations such as arithmetic. This conceptual notion predicts that: 1) SNS are involved in the evolution and development of cognition, and 2) SNS are involved in cognition in species that do not have fully developed cortex (e.g., fish), and 3) SNS are involved in cognitive abilities in the mature human brain even in what is considered “higher-order” cognition, such as arithmetic. We term this conceptual framework the “*SNS hypothesis*.” We propose that SNS, which are ubiquitous across the animal kingdom, enable cognitive operations essential for the emergence of complex cognition (see also^13^). SNS can be reused and manipulated by neocortical mechanisms, and jointly, novel skills can be developed during evolution.

It should be noted that perceptual differences, integration cost, binocular rivalry, and intraocular suppression cannot fully explain the differences in performance observed between the eye-of-origin conditions in the current experiments. First, to preclude any confounding effect of perceptual differences between the eye-of-origin conditions and to determine whether participants experienced a well-fused percept in all the conditions, the stereoscope apparatus was calibrated for each participant individually to ensure perceptual fusion of the images presented in all the conditions (see Methods section for more details). Second, both in the solution to a different-eye condition and in the computational term split condition, one of the numbers in the equation was presented to a different eye. If presenting one number to a different eye hampers performance, regardless of the involvement of a symbolic computational process, performance should be impaired in the solution to a different-eye condition compared with the all in one-eye condition. This was not the case in all four experiments. Third, when looking at the computational term split condition, there was no significant difference between equations in which the number presented to the different eye was from the first or third location. This indicates that the spatial location of the number that was presented to the other eye cannot fully explain the findings. Lastly, the same perceptual factors are involved both in equations containing single-digit numbers and in equations containing double-digit numbers. In Experiment 4, which included double-digit numbers, there was no difference in performance between the eye-of-origin conditions (in contrast to Experiments 1–3), indicating that the stereoscope manipulation by itself cannot account for the findings in the first three experiments. Singly and collectively, the above-mentioned considerations render alternative, nonnumerical explanations for the observed differences unlikely.

It should also be noted that solving arithmetic equations might involve memory-based retrieval processes and not only arithmetic calculations. However, the current pattern of results cannot be fully explained only by memory processes. It is widely accepted that young children's performance on arithmetic tasks is often based on counting or other procedural strategies, although some memory-retrieval processes can be found for small problems such as 2 + 2^62^. However, how we mentally represent and process basic arithmetic such as 5 + 7 has been debated for over three decades^63,64^. While multiplication rely on memory processes, subtraction and addition, as used in our experiments, are considered to rely more on computational processes/arithmetic reasoning^41,43,65^. Several studies demonstrated that simple addition of two-digits equations is not exclusively based on memory-retrieval and does require computational processes^43,66^. This is also true for subtraction^67^. In addition, Sklar et al. (2012) report that 3 term addition and subtraction take roughly a 1000 ms more than two-term addition/subtraction. This is highly indicative of a computation. Accordingly, the arithmetic equations employed in Experiment 1 and Experiment 2 were three-digit equations (e.g., 4 + 3 + 8 = 15).

Indeed, we are unaware of any indications in the literature that such equations can be solved using arithmetical fact retrieval alone. Hence, the possibility that memory retrieval processes could account for the results of Experiment 1 and Experiment 2 is unlikely. Moreover, most of the equations used in Experiment 1, Experiment 2, and Experiments 3 required double-digit complex arithmetic calculations (e.g., sum of 18) which involves computational/arithmetic reasoning. Since solving three-digit equations requires using computational processes, we believe that our findings reflect the involvement of subcortical regions in computationally demanding arithmetic reasoning processes. In addition, in Experiment 1 and Experiment 2, both in the solution to a different-eye condition and in the computational term split condition, one of the numbers in the equation was presented to a different eye. In both conditions, presenting one number to a different eye should hamper memory processes to the same extent. This was not evident in Experiment 1 and Experiment 2, in which performance was hampered only in the computational term split condition. Hence, the current pattern of results cannot be fully explained only by memory-retrieval processes, and some computational processes should be involved. This convergent evidence makes the memory explanation less plausible.

One remaining question is which specific lower visual region is involved in arithmetic abilities. The three main candidates of the visual system are V1, thalamus, and the superior colliculus (SC), but the proposed method and logic do not allow to localize the specific subcortical region involved in those abilities. However, recent studies have suggested the involvement of the SC in symbolic spatial abilities in humans^10^ and fish^55^, and even in numerical processes^22^. It is possible that the SC is also involved in more complex symbolic arithmetic processes. Even when V1 is not activated, visual input from the SC can activate the dorsal visual stream^68,69^. The direct connection of the SC to parietal regions supports a functional relationship between these regions and may suggest the SC as a favorite candidate through which subcortical regions are involved in arithmetic calculations.

To conclude, in contrast to most literature, research conducted in recent years has taught us that many of the high-level functions, which were traditionally associated with neocortical regions, can functionally involve lower subcortical regions^10,13,14,22,37^. The current findings demonstrate that a uniquely human cultural product, such as solving arithmetic equations, does not solely involve neocortical regions, and they suggest a primitive mechanism for arithmetic abilities that might be shared by different species. As we have discussed, the results may have major implications for our understanding of the neuroevolutionary development of numerical abilities in general. Finally, these results suggest that the modal view of higher cognition and lower cognition, a view that ties together humans’ unique neocortical regions with humans’ unique (at least as assumed in the literature) arithmetic capacities, should be significantly updated. In a larger conceptual framework, these findings, and others, call for a significant shift from the modal view of the exclusive role of the neocortex in high-level cognition and arithmetic processes to a view that emphasizes the interplay between subcortical and cortical brain mechanisms.

## Methods

### Participants

One hundred forty-three students (109 female, mean age = 22.98) participated in 4 experiments for payment or course credit: 45 participants in Experiment 1, 33 participants in Experiment 2, 33 participants in Experiment 3, 32 participants in Experiment 4. We conducted a power analysis using G*Power 3.1^70^ to assess the sample size required for testing our eye-of-origin effects. As far as we know, there are no previous studies that used a similar stereoscopic manipulation in arithmetic processes. Hence, we examined previous studies that used a stereoscopic manipulation to examine different cognitive abilities (i.e., attention and numerical abilities), or studies that examine arithmetic abilities but without the stereoscopic manipulation. Previous studies that have used a similar stereoscopic manipulation revealed small to medium effect sizes (e.g., Saban, Gabay, et al., 2017, ${\upeta }_{\mathrm{p}}^{2}$= 0.08; Collins et al., 2017, ${\upeta }_{\mathrm{p}}^{2}$= 0.01). Hence, in a strict manner, a small effect size was expected in the present experiments (${\upeta }_{\mathrm{p}}^{2}=.01)$. Given the lowest observed correlation between repeated samples in the current set of experiments (r = 0.95), the analysis revealed that for a power = 90% with α = 0.05, we would need 28 participants. Therefore, the sample sizes of the present studies (i.e., minimum 32) were sufficiently powered. In addition, our sample size in each experiment was in accordance with previous studies in the field of arithmetic^6,22,72^, that is, with the same or a lower sample size in previous studies (sample between 17 and 31). Finally, a sensitivity analysis of the critical comparisons in our experiments revealed that the sample sizes (> 31) were sufficiently sensitive to detect small effects (${\upeta }_{\mathrm{p}}^{2}$> 0.008) with 90% power.

In all experiments, participants had normal or corrected-to-normal vision and no history of attention deficit or learning disabilities, average RTs of correct responses (> 90% of all trials) in each task were calculated, participants were excluded from the analyses if they performed worse than chance level ($\le$ 50% accuracy rate; less than 5% of all participants), and trials in which RTs were very low or very high were excluded (RT < 200 ms & RT > 4000 ms; less than 5% of all trials). The study was approved by the University of Haifa ethics committee and the experiments were performed in accordance with relevant guidelines and regulations. Informed consent was obtained from all participants.

### Stimuli and experimental design

Stimulus presentation was performed using a HP Z200 computer, operating with a Windows 7 system. The stimuli were displayed on a Samsung LCD monitor (model S24C650PL) with a resolution of 1,680 × 1,050. Responses were made using a DELL Hebrew–English Extended Keyboard (model RT7D50 SK-8115). The computer monitor was positioned 57 cm in front of a stereoscope (model ScreenScope LCD SA200LCD), blocking the participant’s direct view of the monitor. The monitor presentation was divided into two halves (each half was presented to a different eye) and consisted of two rectangles (4.8° in width and 14.2° in height), placed 8.5° from the center of the screen and 16.7° from each other. A central fixation cross composed of two lines (1° width and height) was presented to both eyes. Each equation, placed in one of the rectangles, was then presented to one of the eyes. All stimuli were white figures against a black background.

A similar procedure was used in all the experiments. In Experiment 1, each experimental trial began with a fixation rectangle appearing for 1 s to both eyes. Afterwards, an equation consisting of three single-digit numbers on the left-hand side of the equal sign and the solution on the right-hand side were presented for 5 s or until response. The participants completed a verification task, in which they were instructed to press the “B” button if the equation was correct and the “N” button if the equation was incorrect as quickly and as accurately as they could. In Experiments 1 and 2, we manipulated the incorrect answers’ distance from the correct answer since responding to an equation with a small distance requires exact calculation, whereas responding to an equation with a bigger distance relies on rough estimation to some extent. In the addition equations, the distance from the correct answer was 0, 2, and 4 and for the subtraction equations the distance was 0, 1, and 3. For instance, a distance of 2 for the sum of 4 + 3 = 9 means that the presented sum was 9 but the correct sum was 7. Each equation was matched with one correct solution and one incorrect solution. No feedback was given.

The equations were displayed in three eye-of-origin conditions: (1) all in one-eye: the arithmetic problem (i.e., the three single-digit numbers on the left-hand side of the equal sign) and the solution were displayed to the same eye; (2) solution to a different-eye: the arithmetic problem and the solution were each displayed to a different eye; (3) Computational term split: one of the three numbers of the arithmetic problem was displayed to one eye and the other two numbers and the solution were displayed to the other eye. In the third condition, the digit that was presented to a different eye was equally chosen for all three possible locations. The experiment consisted of two blocks, one containing addition equations and one containing subtraction equations, which were presented in a random and counterbalanced design. After the participant responded, the equation disappeared for 2 s. This was followed by the presentation of the blank rectangles for 1 s before the beginning of the next trial (ITI). Each participant completed 16 practice trials in each block (one block for addition and one block for subtraction) and 192 experimental trials in each block (for a total of 416 trials). For each Eye-of-Origin condition, there were 32 trials of correct equations (distance = 0) and 32 of incorrect Eqs. (16 trials for each non-zero distance). The trials were randomly presented and counterbalanced for correctness and eye-of-origin.

For each participant, the stereoscope apparatus was calibrated to ensure perceptual fusion of the images presented in all the conditions^12,13,37,60^. Before the experiment began, we conducted two tests to preclude any confounding effect of perceptual differences between the eye-of-origin conditions and to determine whether participants experienced a well-fused percept in all the conditions^12,13,37,60^. Two rectangles were presented throughout the task, one to each eye, and all stimuli were presented inside those rectangles^12,13,37,60^. First, we asked each participant whether they saw a single rectangle or two overlapping rectangles when looking through the stereoscope. If a participant reported seeing two overlapping rectangles the stereoscope was calibrated in order to achieve a fused percept of a single rectangle. Second, each participant was also instructed to close one eye at a time and asked if they saw a full rectangle to make sure that the visual display was full for each eye separately^12,13,37,60^. If a participant reported seeing only a part of the rectangle, the stereoscope was recalibrated. These tests ensured that the percept was well fused during the task in all conditions.

## References

[1] Grabner RH (2009). To retrieve or to calculate? Left angular gyrus mediates the retrieval of arithmetic facts during problem solving. Neuropsychologia.

[2] Dehaene S, Cohen L (1997). Cerebral pathways for calculation: Double dissociation between rote verbal and quantitative knowledge of arithmetic. Cortex.

[3] Dehaene S, Piazza M, Pinel P, Cohen L (2003). Three parietal circuits for number processing. Cogn. Neuropsychol..

[4] Dehaene S, Molko N, Cohen L, Wilson AJ (2004). Arithmetic and the brain. Curr. Opin. Neurobiol..

[5] Andres M, Pelgrims B, Michaux N, Olivier E, Pesenti M (2011). Role of distinct parietal areas in arithmetic: An fMRI-guided TMS study. Neuroimage.

[6] Ansari D, Dhital B (2006). Age-related changes in the activation of the intraparietal sulcus during nonsymbolic magnitude processing: An event-related functional magnetic resonance imaging study. J. Cogn. Neurosci..

[7] Arsalidou M, Taylor MJ (2011). Is 2 + 2 = 4? Meta-analyses of brain areas needed for numbers and calculations. Neuroimage.

[8] Emerson RW, Cantlon JF (2015). Continuity and change in children’s longitudinal neural responses to numbers. Dev. Sci..

[9] Dahlin E, Neely AS, Larsson A, Backman L, Nyberg L (2008). Transfer of learning after updating training mediated by the striatum. Science.

[10] Saban, W., Sekely, L., Klein, R. M. & Gabay, S. Monocular channels have a functional role in endogenous orienting. *Neuropsychologia* **111**, (2018). 10.1016/j.neuropsychologia.2018.01.002 29317323

[11] Saban, W., Weinbach, N. & Gabay, S. Monocular channels have a functional role in phasic alertness and temporal expectancy. *Attention, Perception, Psychophys.* (2019). 10.3758/s13414-018-01653-9 10.3758/s13414-018-01653-9 30628033

[12] Saban W, Gabay S, Kalanthroff E (2018). More than just channeling: The role of subcortical mechanisms in executive functions—Evidence from the Stroop task. Acta Psychol. (Amst).

[13] Saban W, Raz G, Grabner RH, Gabay S, Kadosh RC (2021). Primitive visual channels have a causal role in cognitive transfer. Sci. Rep..

[14] Salminen XT, Ku S, Frensch PA, Schubert T (2016). Transfer after dual n -back training depends on striatal activation change. J. Neurosci..

[15] Parvizi J (2009). Corticocentric myopia: old bias in new cognitive sciences. Trends Cogn. Sci..

[16] LaBar KS, Gitelman DR, Mesulam MM, Parrish TB (2001). Impact of signal-to-noise on functional MRI of the human amygdala. NeuroRep..

[17] Dehaene S, Cohen L (2007). Cultural recycling of cortical maps. Neuron.

[18] Dehaene S (2011). The Number Sense: How the Mind Creates Mathematics.

[19] Adams, J. W., Barmby, P. & Mesoudi, A. *The Nature and Development of Mathematics: Cross Disciplinary Perspectives on Cognition, Learning and Culture*. *The Nature and Development of Mathematics: Cross Disciplinary Perspectives on Cognition, Learning and Culture* (Taylor and Francis, 2017). 10.4324/9781315648163

[20] Starr A, Libertus ME, Brannon EM (2013). Number sense in infancy predicts mathematical abilities in childhood. Proc. Natl. Acad. Sci. U. S. A..

[21] Halberda, J., Ly, R., Wilmer, J. B., Naiman, D. Q. & Germine, L. Number sense across the lifespan as revealed by a massive Internet-based sample. 10.1073/pnas.1200196109 10.1073/pnas.1200196109 PMC3396479 22733748

[22] Collins E, Park J, Behrmann M (2017). Numerosity representation is encoded in human subcortex. Proc. Natl. Acad. Sci..

[23] Nieder, A. A brain for numbers : the biology of the number instinct. 376

[24] Agrillo C, Piffer L, Bisazza A (2010). Large number discrimination by mosquitofish. PLoS ONE.

[25] Pepperberg IM (2006). Grey parrot numerical competence: a review. Anim. Cogn..

[26] Gross HJ (2009). Number-based visual generalisation in the honeybee. PLoS ONE.

[27] Nelson XJ, Jackson RR (2012). The role of numerical competence in a specialized predatory strategy of an araneophagic spider. Anim. Cogn..

[28] Rose GJ (2017). The numerical abilities of anurans and their neural correlates: insights from neuroethological studies of acoustic communication. Philos. Trans. R. Soc. Lond. B. Biol. Sci..

[29] Howard SR, Avarguès-Weber A, Garcia JE, Greentree AD, Dyer AG (2018). Numerical ordering of zero in honey bees. Science.

[30] Rugani R, Regolin L, Vallortigara G (2008). Discrimination of small numerosities in young chicks. J. Exp. Psychol. Anim. Behav. Process..

[31] Rugani R, Fontanari L, Simoni E, Regolin L, Vallortigara G (2009). Arithmetic in newborn chicks. Proc. R. Soc. B Biol. Sci..

[32] Rugani R, Vallortigara G, Regolin L (2016). Mapping number to space in the two hemispheres of the avian brain. Neurobiol. Learn. Mem..

[33] Lorenzi, E., Perrino, M. & Vallortigara, G. Numerosities and Other Magnitudes in the Brains: A Comparative View. *Front. Psychol.*, p. 1104 (2021) 10.3389/fpsyg.2021.641994 PMC8082025 33935896

[34] Menon RS, Ogawa S, Strupp JP, Uǧurbil K (1997). Ocular dominance in human V1 demonstrated by functional magnetic resonance imaging. J. Neurophysiol..

[35] Horton JC, Dagi LR, McCrane EP, de Monasterio FM (1990). Arrangement of ocular dominance columns in human visual cortex. Arch. Ophthalmol. Chicago Ill.

[36] Batson MA, Beer AL, Seitz AR, Watanabe T (2011). Spatial shifts of audio-visual interactions by perceptual learning are specific to the trained orientation and eye. Seeing Perceiving.

[37] Saban, W., Klein, R. M. & Gabay, S. Probabilistic versus “Pure” Volitional Orienting: a Monocular Difference. *Attention, Perception, Psychophys.* **80**, (2018). 10.3758/s13414-017-1473-8 29322317

[38] De Smedt B, Holloway ID, Ansari D (2011). Effects of problem size and arithmetic operation on brain activation during calculation in children with varying levels of arithmetical fluency. Neuroimage.

[39] Dienes Z (2014). Using Bayes to get the most out of non-significant results. Front. Psychol..

[40] Lee MD, Wagenmakers EJ (2013). Bayesian cognitive modeling: a practical course. Bayesian Cogn. Model. A Pract. Course.

[41] Campbell JID, Xue Q (2001). Cognitive arithmetic across cultures. J. Exp. Psychol. Gen..

[42] Dehaene S (1992). Varieties of numerical abilities. Cognition.

[43] LeFevre JA, Sadesky GS, Bisanz J (1996). Selection of procedures in mental addition: reassessing the problem size effect in adults. J. Exp. Psychol. Learn. Mem. Cogn..

[44] Ashcraft MH, Guillaume MM (2009). Chapter 4 mathematical cognition and the problem size effect. Psychol. Learn. Motiv. Adv. Res. Theory.

[45] Aarsen FK, Arts WFM, Veelen-Vincent MLC, Van Lequin MH, Catsman-Berrevoets CE (2014). Original article Long-term outcome in children with low grade tectal tumours and obstructive hydrocephalus. Eur. J. Paediatr. Neurol..

[46] Delazer M (2004). Number processing and basal ganglia dysfunction: a single case study. Neuropsychologia.

[47] Ojemann, G. A. Mental arithmetic during human thalamic stimulation*. **12**, (1974). 10.1016/0028-3932(74)90021-9 4595224

[48] Leibovich T, Katzin N, Harel M, Henik A (2017). From “sense of number” to “sense of magnitude”: The role of continuous magnitudes in numerical cognition. Behav. Brain Sci..

[49] Gabay S, Leibovich T, Henik A, Gronau N (2013). Size before numbers: Conceptual size primes numerical value. Cognition.

[50] Kutter EF, Bostroem J, Elger CE, Mormann F, Correspondence AN (2018). Single neurons in the human brain encode numbers. Neuron.

[51] Gallistel, C. R., & Gelman, R. *Mathematical Cognition*. (Cambridge University Press., 2005).

[52] Kanjlia S, Feigenson L, Bedny M (2018). Numerical cognition is resilient to dramatic changes in early sensory experience. Cognition.

[53] Sklar, A. Y. *et al.* Reading and doing arithmetic nonconsciously. **109**, (2012). 10.1073/pnas.1211645109 PMC3511729 23150541

[54] Saban W, Sekely L, Klein RM, Gabay S (2018). Monocular channels have a functional role in endogenous orienting. Neuropsychologia.

[55] Saban W, Sekely L, Klein RM, Gabay S (2017). Endogenous orienting in the archer fish. Proc. Natl. Acad. Sci..

[56] Messina A (2020). Response to change in the number of visual stimuli in zebrafish: A behavioural and molecular study. Sci. Rep..

[57] Leibovich-Raveh T, Raveh A, Vilker D, Gabay S (2021). Magnitude integration in the Archerfish. Sci. Rep..

[58] Soloveichick M, Kimchi R, Gabay S (2021). Functional involvement of subcortical structures in global-local processing. Cognition.

[59] Saban, W., Weinbach, N. & Gabay, S. Monocular channels have a functional role in phasic alertness and temporal expectancy. *Attention, Perception, Psychophys.* **81**, (2019). 10.3758/s13414-018-01653-9 30628033

[60] Saban W, Weinbach N, Gabay S (2019). Monocular channels have a functional role in phasic alertness and temporal expectancy. Attention Perception Psychophys..

[61] Anderson, J. R. *How Can the Human Mind Occur in the Physical Universe? How Can the Human Mind Occur in the Physical Universe?* (2007). doi:10.1093/acprof:oso/9780195324259.001.0001

[62] Siegler, R. S. & Shrager, J. Strategy choices in addition and subtraction: How do children know what to do? *Orig. Cogn. Ski.* 229–293 (1984).

[63] Ashcraft MH (1982). The development of mental arithmetic: a chronometric approach. Dev. Rev..

[64] Baroody AJ (1994). An evaluation of evidence supporting fact-retrieval models. Learn. Individ. Differ..

[65] LeFevre JA, Morris J (1999). More on the relation between division and multiplication in simple arithmetic: Evidence for mediation of division solutions via multiplication. Mem. Cogn..

[66] Geary DC, Wiley JG (1991). Cognitive addition: Strategy choice and speed-of-processing differences in young and elderly adults. Psychol. Aging.

[67] Geary DC, Frensch PA, Wiley JG (1993). Simple and complex mental subtraction: Strategy choice and speed-of- processing differences in younger and older adults.. Psychol. Aging.

[68] Rodman HR, Gross CG, Albright TD (1989). Afferent basis of visual response properties in area MT of the macaque. I. Effects of striate cortex removal. J. Neurosci..

[69] Rosa MG, Tweedale R, Elston GN (2000). Visual responses of neurons in the middle temporal area of new world monkeys after lesions of striate cortex. J. Neurosci..

[70] Faul F, Erdfelder E, Lang A, Buchner A (2007). G * Power 3: A flexible statistical power analysis program for the social, behavioral, and biomedical sciences. Behav. Res. Methods.

[71] Saban W, Gabay S, Kalanthro E (2017). Acta Psychologica More than just channeling: The role of subcortical mechanisms in executive functions: Evidence from the Stroop task. Acta Physiol. (Oxf).

[72] Grabner RH, De Smedt B (2012). Oscillatory EEG correlates of arithmetic strategies: A training study. Front. Psychol..

